# Associations Between Benzoxazinoids, Plant Development and Nutritional Parameters in Different Maize Maturing Genotypes

**DOI:** 10.1111/jpn.70105

**Published:** 2026-08-14

**Authors:** Julia Eichinger, Jürg Hiltbrunner, Pierre Mateo, Christelle A. M. Robert, Josef J. Gross

**Affiliations:** ^1^ Veterinary Physiology, Vetsuisse Faculty University of Bern Bern Switzerland; ^2^ Agroscope, Competence Division Plant and Plant Products Research Group Extension Arable Crops Zürich Switzerland; ^3^ Laboratory of Chemical Ecology and Health, Faculty of Science, Institute of Plant Sciences University of Bern Bern Switzerland

**Keywords:** maize silage, nutritive value, plant secondary metabolites, ruminants

## Abstract

Maize (*Zea mays* L.) silage is a major energy source in ruminant diets and provides fermentable substrate for the rumen microbiota. In addition, forage maize contains benzoxazinoids (BXs), defence‐related secondary metabolites with antimicrobial activity that remain detectable after ensiling. For ruminant nutrition, it is important to know whether variation in BX is associated with changes in forage nutritive value. This study evaluated 14 commercially relevant forage maize genotypes in dairy‐producing regions, comprising seven early and seven mid‐early maturing genotypes selected from the official Swiss maize trial network. At harvest, plant developmental traits were recorded, nutrient composition was analyzed by near‐infrared spectroscopy, and BX concentrations were quantified by ultra‐high‐performance liquid chromatography–quadrupole time‐of‐flight mass spectrometry (UHPLC–QTOF‐MS). Maturity groups were compared using Wilcoxon rank‐sum tests with Benjamini–Hochberg correction, while associations among variables were assessed using Spearman rank correlations, linear regression models and exploratory principal component analysis (PCA). No significant differences in BX concentrations or nutrient composition were observed between early and mid‐early maturing genotypes (all FDR‐adjusted *p* > 0.05). Consistently, PCA did not indicate a separation between maturity groups, and permutational multivariate analysis of variance showed no significant maturity‐group effect on the multivariate profile (*F* = 1.66, *R* ^2^ = 0.121, *p* = 0.216). Neutral detergent fibre content was negatively correlated with starch concentration (*ρ*_S_ = − 0.70, *P*_S_ = 0.010, *R* ^2^ = 0.66, *P*_lm_ < 0.001), whereas digestible organic matter was correlated with calculated net energy values (*ρ*_S_ ≥ 0.95, *P*_S_ < 0.001, *R* ^2^ ≥ 0.94, *P*_lm_ < 0.001). Several BX metabolite concentrations were negatively correlated with European corn borer incidence, most notably MBOA (*ρ*_S_ = − 0.74, *P*_S_ = 0.002, *R* ^2^ = 0.16, *P*_lm_ = 0.153). Concentrations of DIMBOA and DIMBOA‐Glc were positively associated with net energy for lactation (*ρ*_S_ = 0.64 and 0.68, *P*_S_ = 0.015 and 0.007, *R* ^2^ = 0.32 and 0.34, *P*_lm_ = 0.037 and 0.029, respectively). These findings indicate that plant defence capacity and high forage quality can co‐occur under practical growing conditions.

## 1 Introduction

Maize (*Zea mays* L.) is one of the most important forage crops in ruminant nutrition due to its high biomass yield and energy density. In Switzerland, maize grown for whole‐plant silage is the second most important arable crop after wheat (Buzzi et al. 2021). Its nutritive value is largely influenced by starch and neutral detergent fibre (NDF) content (Khan et al. 2015). These fractions influence digestibility, energy value and the fermentable substrate available to the rumen microbiota and are closely linked to genotype, plant development and harvest maturity (Jensen et al. 2005, Khan et al. 2024). In addition to primary nutritional components, maize contains biologically active secondary metabolites. Among these, benzoxazinoids (BXs) are key defence compounds against herbivores and pathogens (Robert and Mateo 2022). Major BX metabolites, including 2,4‐dihydroxy‐7‐methoxy‐2H‐1,4‐benzoxazin‐3(4H)‐one (DIMBOA) and 2‐β‐d‐glucopyranosyloxy‐4‐hydroxy‐7‐methoxy‐2H‐1,4‐benzoxazin‐3(4H)‐one (DIMBOA‐Glc), are present in green and preserved maize tissues, particularly in leaves and roots, and are regulated by genetic, developmental and environmental factors (Gross et al. 2023, Zhou et al. 2018). The BX are known to influence microbial activity due to their antimicrobial properties and remain detectable after ensiling (Gross et al. 2023). Thus, when maize is used as silage, BX may not only contribute to plant defence before harvest but may also enter ruminant diets as feed‐borne bioactive compounds. From an animal nutrition perspective, it is important to determine whether increased BX concentrations are associated with changes in forage nutritive value. A potential trade‐off between plant defence and forage quality would be relevant for silage‐maize breeding and feeding strategies. Despite this relevance, the relationships between BX accumulation, plant development, agronomic traits and forage quality characteristics remain insufficiently understood. In particular, it remains unclear whether elevated BX concentrations are associated with reductions in key determinants of forage nutritive value, or whether these traits can co‐occur in forage maize grown under practical field conditions. Therefore, the objective of this study was to characterize BX concentrations in different forage maize genotypes and to evaluate their relationships with agronomic traits, plant developmental characteristics and nutrient composition. For the scope of animal nutrition, the study specifically addresses whether BX accumulation is associated with key parameters of forage nutritive value, thereby providing a basis for future studies on feed‐borne BX in ruminant nutrition.

## 2 Materials and Methods

### 2.1 Field Experiment

A field experiment with different maize (*Z. mays* L.) varieties was conducted, where the above‐ground biomass was harvested (date of seeding: May 16, 2017). A total of 60 different genotypes were used in two different trials: containing early and mid‐early maturing genotypes. Both trials were part of the official maize trial network conducted at eight sites in representative growing regions in Switzerland. Details on the site‐specific conditions and the management are published in Agroscope (2017). In brief, maize plants were cultivated under field conditions (individual plots of 10 m^2^ at a plant density of 9.29 ± 0.58 plants/m^2^, 0.75 m row distance) following conventional Swiss farming practices (organic and urea fertilizer, crop rotation, herbicide based on necessity but only once). For the present study, plant material was obtained exclusively from the Grangeneuve site to reduce site‐related environmental variation in BX concentrations and to compare the selected genotypes under identical field conditions.

Grangeneuve is located in a Swiss forage‐maize production region relevant for dairy farming; therefore, its climatic and agronomic conditions are relevant for evaluating maize as ruminant feed under practical Swiss conditions. A subset of seven established early maturing varieties (Schobbi CS, DKC 3333, Kompetens, LG 31.211, Spyci CS, Karibous, SY Amboss) and seven established mid‐early maturing varieties (ES Albatros, SY Talisman, Gottardo KWS, SY Telias, DKC 3440, LG 30.248, LG 31.235) was selected a priori from the official 2017 Swiss silage‐maize trials. These varieties were considered most commercially relevant for Swiss forage production in dairy‐producing regions. Individual genotype data for agronomic traits and nutrient composition of all genotypes and all eight sites are available in the official Agroscope maize variety trial report (Agroscope 2017). Early maturing varieties were harvested on September 26, 2017, corresponding to 133 days after sowing, whereas mid‐early maturing varieties were harvested on October 4, 2017 (141 days after sowing). The average temperature from May to October 2017 was 17°C ± 3°C and cumulative precipitation amounted to 425 mm (average from May to October 2017: 70 ± 28 mm; MeteoSwiss). The following agronomic traits were recorded as described by Agroscope (2017): Early vigour, days to silking (female flowering), days to anthesis (male flowering), plant height, ear insertion height, incidence of common smut, incidence of European corn borer (*Ostrinia nubilalis*), effective plant density and fresh matter yield. At harvest, maize plants per variety were cut at 15 cm above‐ground level as pooled samples per genotype and immediately transported in plastic bags to the research station of Agroscope, Posieux, Switzerland, where they were chopped into pieces with a particle length of 1–2 cm with a modified forage cutter (MEX GT, Poettinger, Grieskirchen, Austria) and stored at −80°C until analysis of nutrient composition and BX concentrations.

### 2.2 Analysis of Nutrient Composition and BX Concentrations

Feed samples were processed and analyzed by near‐infrared spectroscopy (NIRS) for their nutrient composition in triplicate at the ISO 17025‐accredited feed laboratory of Agroscope, Posieux, Switzerland (STS0077; Swiss Accreditation Service [SAS] 2024). The dry matter (DM) content was determined in chopped forage samples dried at 60°C for 20 h and then at 105°C for 3 h as described in Gross et al. (2023). Dried subsamples were ground to pass a 1.0‐mm sieve (Brabender mill with titanium blades; Brabender, Duisburg, Germany) before analysis of their ash, NDF, starch and crude protein (CP) contents. The NIRS calibrations were based on certified reference analyses and validated according to the procedures described by Ampuero Kragten and Wyss (2014). Contents of net energy for lactation (NEL) and net energy for fattening (NEV) were calculated according to Agroscope (2021). The concentrations of the BX metabolites were determined using UHPLC‐MS as described earlier (Gross et al. 2023). Briefly, the samples were ground and aliquoted (100 ± 2.5 mg) in 1.5 mL Eppendorf tubes in the presence of liquid nitrogen. Extraction followed with 1000 µL of a 70:30 mixture of methanol (Optima LC/MS Grade, Fisher Scientific AG, Reinach, Switzerland) and ultrapure water (Barnstead GenPure Pro Water Purification System, Fisher Scientific AG, Reinach, Switzerland) containing 0.1% formic acid (Optima LC/MS Grade, Fisher Scientific AG, Reinach, Switzerland), shaking, brief vortex and centrifugation (10°C, 13 Krpm, 20 min). Approximately 750 µL of supernatant was transferred into liquid chromatography (LC) vials and analyzed without further treatment. The analysis was performed with an Acquity i‐Class ultra‐high‐performance liquid chromatography (UHPLC) system equipped with a single quadrupole detector (QDa) with an electrospray source (Waters, USA). Gradient elution was performed on an Acquity BEH C18 column (2.1 × 50 mm i.d., 1.7 mm particle size) at 90%–70% A over 3 min, 70%–60% A over 1 min, 40%–100% B over 1 min, holding at 100% B for 2.5 min, holding at 90% A for 1.5 min (A = 0.1% formic acid/water and B = 0.1% formic acid/acetonitrile). The flow rate was 0.4 mL/min. The column temperature was maintained at 40°C, and the injection volume was 1 µL. The quantification of the compounds was performed using external standard curves (3‐β‐D‐glucopyranosyl‐6‐methoxy‐benzoxazolin‐2(3H)‐one [MBOA‐Glc], N‐(3‐methoxy‐2‐hydroxyphenyl)malonamic acid [HMPMA], 2‐β‐d‐glucopyranosyloxy‐4‐hydroxy‐7‐methoxy‐2H‐1,4‐benzoxazin‐3(4H)‐one [DIMBOA‐Glc], 2‐hydroxy‐7‐methoxy‐2H‐1,4‐benzoxazin‐3(4H)‐one [HMBOA], 2,4‐dihydroxy‐7‐methoxy‐2H‐1,4‐benzoxazin‐3(4H)‐one [DIMBOA], benzoxazolin‐2(3H)‐one [BOA], 2‐β‐D‐glucopyranosyloxy‐4,7‐dimethoxy‐2H‐1,4‐benzoxazin‐3(4H)‐one [HDMBOA‐Glc], 6‐methoxy‐benzoxazolin‐2(3H)‐one [MBOA], 2‐amino‐3H‐phenoxazin‐3‐one [APO], 9‐methoxy‐2‐amino‐3H‐phenoxazin‐3‐one [AMPO] and 9‐methoxy‐2‐acetylamino‐3H‐phenoxazin‐3‐one [AAMPO]). The MBOA and BOA were purchased at Sigma‐Aldrich, Merck & Cie (Shaffhausen, CH). The HMPMA was obtained as a gift from F. A. Macías, Department of Organic Chemistry, Institute of Biomolecules (INBIO), Cadiz, Spain. All the other compounds were obtained through isolation from natural sources or chemical synthesis in our laboratory. When no analytical standard was available, the compound was quantified relative to the closest parent: HMBOA‐Glc and HM2BOA‐Glc were quantified relative to HMBOA, DIM2BOA‐Glc relative to DIMBOA‐Glc, and HDM2BOA‐Glc relative to HDMBOA‐Glc.

### 2.3 Statistical Analysis

Between the two maize maturity groups (early and mid‐early), nonparametric Wilcoxon rank‐sum tests were conducted to assess differences in all assessed agronomic traits, plant nutrient composition parameters and BX metabolite concentrations. Multiple testing correction was applied using the Benjamini–Hochberg false discovery rate (FDR) procedure (MetaboAnalyst; version 6.0). An exploratory principal component analysis (PCA) was performed on BX metabolites, agronomic traits and nutrient composition parameters to visualize multivariate variation among genotypes. Permutational multivariate analysis of variance with 999 permutations was used to test whether the overall multivariate profile differed between early and mid‐early maturing genotypes. In addition, relationships between BX metabolites, agronomic traits and nutrient composition parameters were evaluated in R (version 4.5.2). Specifically, Spearman's rank correlation coefficients (*ρ*_S_), corresponding *p* values (*P*_S_) and 95% confidence intervals were calculated using the cor.test() function from the stats package. Furthermore, simple linear regression models were fitted using the lm() function from the stats package to explore potential linear associations and to obtain complementary *p* values (*P*_lm_). Data visualization and regression plotting were performed using the ggplot2 package. *p* < 0.05 was considered statistically significant, and only those are presented and discussed.

## 3 Results and Discussion

### 3.1 Genotype Effects on Agronomic Traits, Nutrient Composition and BX Concentrations

Early and mid‐early maturing maize genotypes were harvested 133 and 141 days after sowing, respectively, to achieve similar DM contents and enable comparison between maturity groups. The groups did not differ in DM, starch, NDF, CP, digestible organic matter (DOM), or calculated NEL and NEV (all FDR‐adjusted *p* > 0.05; Table 1). If maturity group had differed in physiological development at harvest, the earlier maturing varieties would be expected to show higher DM and starch concentrations and lower NDF concentrations (Jensen et al. 2005; Khan et al. 2024). Thus, the absence of such differences indicates that harvest timing was well planned and implemented, enabling yield and quality to be compared at a similar physiological stage. Consistent with this, no significant differences were observed in BX metabolite concentrations between early and mid‐early genotype groups, although early genotypes showed numerically higher total BX levels (all FDR‐adjusted *p* > 0.05; Table 1). These findings indicate that maturity group alone did not determine forage nutrient composition or BX accumulation under the environmental conditions of the present study. This comparability provides the basis for interpreting subsequent correlation patterns without major confounding effects by maturity stage. This was supported by the PCA showing no separation between early and mid‐early maturing genotypes (*F* = 1.66, *R* ^2^ = 0.121, *p* = 0.216; data not shown). However, the genotypes Kompetens and SY Telias showed greater separation from the remaining genotypes, which is mainly associated with higher concentrations of DIBOA‐Glc and DIMBOA‐Glc (data not shown).

**Table 1:** Agronomic traits, nutrient composition and benzoxazinoid concentrations in early and mid‐early maize genotypes at harvest (*n* = 7 per maize genotype). Values represent means and standard error of the means (SEM) for each genotype group (early and mid‐early).

Parameter	Early	Mid‐early	SEM	FDR
Early vigour (score)	5.69	5.14	0.27	0.306
Days to silking (days after sowing)	61.57	62.14	0.29	0.745
Days to anthesis (days after sowing)	59.34	59.63	0.14	0.883
Plant height (cm)	263.00	276.43	6.71	0.306
Ear insertion height (cm)	103.00	116.71	6.86	0.663
Incidence of common smut (%)	4.33	8.47	2.07	0.663
Incidence of European corn borer damage (%)	0.80	1.51	0.36	0.546
Effective plant density (plants/m^2^)	9.77	8.80	0.49	0.092
Fresh matter yield (dt/ha)	662.51	687.36	12.42	0.663
DM yield (dt/ha)	251.26	249.17	1.04	0.864
DM content (%)	37.94	36.30	0.82	0.546
Digestible organic matter yield (dt/ha)	175.67	172.64	1.51	0.663
Starch yield (dt/ha)	87.56	86.34	0.61	0.883
DOM (g/kg DM)	698.00	692.67	2.66	0.745
Starch (g/kg DM)	347.33	346.00	0.66	0.883
NDF (g/kg DM)	370.29	378.23	3.97	0.663
Crude protein (g/kg DM)	66.89	65.51	0.69	0.663
NEL (MJ/kg DM)	6.30	6.29	0.01	0.969
NEV (MJ/kg DM)	6.49	6.41	0.04	0.745
MBOA (µg/g DM)	2.57	2.09	0.24	0.663
HDMBOA‐Glc (µg/g DM)	8.08	4.76	1.66	0.663
DIMBOA‐Glc (µg/g DM)	53.42	45.81	3.81	0.745
DIMBOA (µg/g DM)	11.11	10.51	0.30	0.663
DIM2BOA‐Glc (µg/g DM)	32.33	30.84	0.75	0.663
MBOA‐Glc (µg/g DM)	0.19	0.22	0.02	0.663
HBOA‐Glc (µg/g DM)	22.74	23.22	0.24	1.000
DIBOA‐Glc (µg/g DM)	320.70	193.82	63.44	0.663
HMBOA‐Glc (µg/g DM)	27.21	19.45	3.88	0.663
HM2BOA‐Glc (µg/g DM)	1.39	1.12	0.13	0.663
HDM2BOA‐Glc (µg/g DM)	1.23	1.10	0.07	0.943
Total benzoxazinoids (µg/g DM)	480.96	332.92	74.02	0.663

*Note:* Differences between genotype groups were assessed using Wilcoxon signed‐rank tests with Benjamini–Hochberg (FDR) correction. Adjusted *p* < 0.05 was considered statistically significant.

Abbreviations: BX, benzoxazinoids on DM basis; CP, crude protein; DIMBOA, 2,4‐dihydroxy‐7‐methoxy‐2H‐1,4‐benzoxazin‐3(4H)‐one; DIMBOA‐Glc, 2‐β‐d‐glucopyranosyloxy‐4‐hydroxy‐7‐methoxy‐2H‐1,4‐benzoxazin‐3(4H)‐one; DM, dry matter; DOM, digestible organic matter; FDR, false discovery rate; NDF, neutral detergent fibre; NEL, net energy for lactation; NEV, net energy for fattening.

### 3.2 Relationships Among Agronomic Traits

To investigate how agronomic traits, nutrient composition and BX accumulation in maize were associated in the present study, Spearman rank correlations were evaluated (Table 2). Days from sowing to anthesis and days to silking, respectively, were strongly correlated (*ρ*_S_ = 0.82, *P*_S_ < 0.001, *R* ^2^ = 0.72, *P*_lm_ < 0.001), consistent with the close temporal coordination of male and female flowering processes during maize reproductive development. Plant height was positively associated with ear insertion height (*ρ*_S_ = 0.62, *P*_S_ = 0.019, *R* ^2^ = 0.53, *P*_lm_ = 0.003), indicating that taller genotypes tended to have higher ear placement. Moreover, ear height was positively correlated with days to anthesis (*ρ*_S_ = 0.59, *P*_S_ = 0.025, *R* ^2^ = 0.25, *P*_lm_ = 0.071) and with days to silking (*ρ*_S_ = 0.67, *P*_S_ = 0.014, *R* ^2^ = 0.25, *P*_lm_ = 0.066). This pattern is consistent with the previously described coupling between phenological timing and biomass partitioning in maize, where longer vegetative duration often allows greater stem elongation and affects ear placement (Du et al. 2021). Plant density was negatively correlated with European corn borer incidence (*ρ*_S_ = − 0.59, *P*_S_ = 0.026, *R* ^2^ = 0.26, *P*_lm_ = 0.063), potentially indicating that stand density may influence herbivore pressure, potentially through altered microclimatic conditions or host accessibility (Oyediran et al. 2016).

**Table 2:** Significant Spearman rank and linear correlations between agronomic traits, nutrient composition and benzoxazinoid metabolite concentrations across maize genotypes.

Parameter 1	Parameter 2	*ρ*_S_	*R* ^2^	95% CI_S_	*P*_S_	*P*_lm_
*Agronomic traits*						
Days to anthesis (days)	Days to silking (days)	0.82	0.72	0.41, 0.97	< 0.001	< 0.001
Plant height (cm)	Maize ear height (cm)	0.62	0.53	0.02, 0.94	0.019	0.003
Maize ear height (cm)	Days to anthesis (days)	0.59	0.25	0.10, 0.89	0.025	0.071
Maize ear height (cm)	Days to silking (days)	0.67	0.25	0.25, 0.88	0.014	0.066
Plant density (plants/m^2^)	Corn borer (%)	−0.59	0.26	−0.82, −0.09	0.026	0.063
*Nutrient composition*						
NDF (g/kg DM)	Starch (g/kg DM)	−0.70	0.66	−0.93, −0.21	0.010	< 0.001
NEL (MJ/kg DM)	DOM (g/kg DM)	0.97	0.94	0.90, 0.99	< 0.001	< 0.001
NEV (MJ/kg DM)	DOM (g/kg DM)	0.95	0.96	0.86, 0.99	< 0.001	< 0.001
Starch (g/kg DM)	DOM (g/kg DM)	0.55	0.48	−0.06, 0.88	0.043	0.006
Starch (g/kg DM)	NEL (MJ/kg DM)	0.58	0.43	0.08, 0.88	0.031	0.011
Starch yield (kg/ha)	NEV (MJ/kg DM)	0.64	0.59	0.10, 0.94	0.014	< 0.001
CP (g/kg DM)	NDF (g/kg DM)	−0.67	0.59	−0.94, −0.19	0.008	< 0.001
CP (g/kg DM)	DOM (g/kg DM)	0.61	0.66	0.03, 0.95	0.019	< 0.001
CP (g/kg DM)	NEL (MJ/kg DM)	0.60	0.49	−0.10, 0.92	0.024	0.005
CP (g/kg DM)	NEV (MJ/kg DM)	0.75	0.67	0.36, 0.94	0.002	< 0.001
CP (g/kg DM)	DM yield (kg/ha)	0.65	0.30	0.15, 0.90	0.013	0.043
*Benzoxazinoid concentrations*						
BX (µg/g DM)	Early vigour (score)	0.56	0.15	0.04, 0.89	0.036	0.173
MBOA (µg/g DM)	Corn borer (%)	−0.74	0.16	−0.95, −0.27	0.002	0.153
HMBOA‐Glc (µg/g DM)	Corn borer (%)	−0.68	0.27	−0.92, −0.14	0.007	0.059
DIMBOA (µg/g DM)	Corn borer (%)	−0.59	0.10	−0.90, −0.13	0.025	0.278
DIMBOA‐Glc (µg/g DM)	Corn borer (%)	−0.54	0.14	−0.90, −0.06	0.044	0.191
DIM2BOA‐Glc (µg/g DM)	Corn borer (%)	−0.61	0.11	−0.92, −0.15	0.022	0.243
DIMBOA (µg/g DM)	DOM (g/kg DM)	0.59	0.24	0.09, 0.88	0.025	0.076
DIMBOA‐Glc (µg/g DM)	DOM (g/kg DM)	0.61	0.27	0.09, 0.86	0.022	0.057
DIMBOA (µg/g DM)	NEL (MJ/kg DM)	0.64	0.32	0.15, 0.92	0.015	0.037
DIMBOA‐Glc (µg/g DM)	NEL (MJ/kg DM)	0.68	0.34	0.31, 0.84	0.007	0.029
DIMBOA (µg/g DM)	NEV (MJ/kg DM)	0.56	0.23	0.11, 0.89	0.035	0.083
DIMBOA‐Glc (µg/g DM)	NEV (MJ/kg DM)	0.64	0.26	0.25, 0.83	0.015	0.064

*Note:* Spearman correlation coefficients (*ρ*_S_), coefficient of determination from the linear regression model (*R* ^2^), 95% confidence intervals of Spearman correlations (CI_S_), Spearman *p* values (*P*_S_), linear model *p* values (*P*_lm_). *p* < 0.05 was considered statistically significant.

Abbreviations: BX, benzoxazinoids on DM basis; CP, crude protein; DIMBOA, 2,4‐dihydroxy‐7‐methoxy‐2H‐1,4‐benzoxazin‐3(4H)‐one; DIMBOA‐Glc, 2‐β‐d‐glucopyranosyloxy‐4‐hydroxy‐7‐methoxy‐2H‐1,4‐benzoxazin‐3(4H)‐one; DM, dry matter; DOM, digestible organic matter; NDF, neutral detergent fibre; NEL, net energy for lactation; NEV, net energy for fattening.

### 3.3 Relationships Among Nutrient Composition

Strong relationships were further observed among nutrient composition parameters (Table 2). The NDF concentration was negatively correlated with starch concentration (*ρ*_S_ = − 0.70, *P*_S_ = 0.010, *R* ^2^ = 0.66, *P*_lm_ < 0.001), consistent with genotypic variation in biomass allocation between structural cell wall components and starch‐rich grain tissue, which are key determinants of forage digestibility and energy density in maize (Jensen et al. 2005; Khan et al. 2024). Accordingly, the calculated energy‐related parameters NEL and NEV were both strongly correlated with DOM concentrations (*ρ*_S_ = 0.97 and 0.95, respectively; both *P*_S_ < 0.001, *R* ^2^ = 0.94 and 0.96, respectively; both *P*_lm_ < 0.001). Starch concentration was positively correlated with DOM concentration (*ρ*_S_ = 0.55, *P*_S_ = 0.043, *R* ^2^ = 0.48, *P*_lm_ < 0.006), with NEL (*ρ*_S_ = 0.58, *P*_S_ = 0.031, *R* ^2^ = 0.43, *P*_lm_ = 0.011) and NEV (*ρ*_S_ = 0.64, *P*_S_ = 0.014, *R* ^2^ = 0.59, *P*_lm_ < 0.001). These relationships support the relevance of starch accumulation for forage energy value under comparable harvest maturity (Jensen et al. 2005; Khan et al. 2024). Interestingly, CP concentration was negatively associated with NDF (*ρ*_S_ = −0.67, *P*_S_ < 0.008, *R* ^2^ = 0.59, *P*_lm_ < 0.001) and positively associated with DOM (*ρ*_S_ = 0.61, *P*_S_ = 0.019, *R* ^2^ = 0.66, *P*_lm_ < 0.001), the energy content (NEL, *ρ*_S_ = 0.60, *P*_S_ = 0.024, *R* ^2^ = 0.49, *P*_lm_ = 0.005; NEV, *ρ*_S_ = 0.75, *P*_S_ < 0.002, *R* ^2^ = 0.67, *P*_lm_ < 0.001) and DM yield (*ρ*_S_ = 0.65, *P*_S_ = 0.013, *R* ^2^ = 0.30, *P*_lm_ = 0.043). This pattern differs from the classical maturity‐related dilution effect in maize, where CP concentration typically declines with advancing maturity due to dilution by starch accumulation and increased grain proportion (Khan et al. 2024). However, in the present study, DM concentration and starch levels did not differ systematically across genotypes, which may indicate comparable physiological maturity at harvest. Therefore, the observed variation in CP concentration may be related to differences in nitrogen availability, uptake efficiency, or plant vigour.

### 3.4 Relationships of BX Concentrations With Agronomic Traits and Nutrient Composition

The BX concentrations in maize plants were associated with selected plant developmental traits and insect pressure (Table 2). Early vigour was positively correlated with total BX concentration at harvest (*ρ*_S_ = 0.56, *P*_S_ = 0.036, *R* ^2^ = 0.15, *P*_lm_ = 0.173), indicating that plants with more advanced or vigorous early development may show higher total BX concentrations at harvest. This association is consistent with previous reports linking secondary metabolite profiles such as BX with plant physiological activity, growth performance and stress tolerance (Hu et al. 2021; Zhou et al. 2018). In addition to developmental regulation, several BX metabolites were negatively correlated with European corn borer incidence. Specifically, negative correlations were observed for MBOA (*ρ*_S_ = − 0.74, *P*_S_ = 0.002, *R* ^2^ = 0.16, *P*_lm_ = 0.153), HMBOA‐Glc (*ρ*_S_ = − 0.68, *P*_S_ = 0.007, *R* ^2^ = 0.27, *P*_lm_ = 0.059), DIMBOA (*ρ*_S_ = − 0.59, *P*_S_ = 0.025, *R* ^2^ = 0.10, *P*_lm_ = 0.278), DIMBOA‐Glc (*ρ*_S_ = − 0.54, *P*_S_ = 0.044, *R* ^2^ = 0.14, *P*_lm_ = 0.191) and DIM2BOA‐Glc (*ρ*_S_ = − 0.61, *P*_S_ = 0.022, *R* ^2^ = 0.11, *P*_lm_ = 0.243). These relationships are consistent with the established role of BX in plant defence against herbivorous insects (Robert and Mateo 2022; Zhou et al. 2018) and with previous evidence that European corn borer preferentially selects plants with lower BX concentrations (Maag et al. 2014). Importantly for ruminant nutrition, BX concentrations were not negatively associated with forage quality. Instead, DIMBOA and DIMBOA‐Glc concentrations were positively correlated with DOM (*ρ*_S_ = 0.59, *P*_S_ = 0.025, *R* ^2^ = 0.24, *P*_lm_ = 0.076; *ρ*_S_ = 0.61, *P*_S_ = 0.022, *R* ^2^ = 0.27, *P*_lm_ = 0.057, respectively), NEL (*ρ*_S_ = 0.64, *P*_S_ = 0.015, *R* ^2^ = 0.32, *P*_lm_ = 0.037; *ρ*_S_ = 0.68, *P*_S_ = 0.007, *R* ^2^ = 0.34, *P*_lm_ = 0.029, respectively) and NEV (*ρ*_S_ = 0.56, *P*_S_ = 0.035, *R* ^2^ = 0.23, *P*_lm_ = 0.083; *ρ*_S_ = 0.64, *P*_S_ = 0.015, *R* ^2^ = 0.26, *P*_lm_ = 0.064, respectively). Thus, higher BX concentrations were not associated with lower digestibility or the calculated energy value in the present forage maize genotypes. From a feed evaluation perspective, this finding suggests that plant defence‐related secondary metabolites and favourable nutritive value can co‐occur at comparable silage‐maize harvest maturity, rather than indicating an apparent trade‐off between chemical defence and feed value (Hu et al. 2021; Zhou et al. 2018). From a ruminant nutrition perspective, these findings are relevant because maize silage is a major dietary component and BX may be ingested as feed‐borne bioactive compounds. BXs have antimicrobial properties and can modulate microbial activity in plant‐associated ecosystems (Zhou et al. 2018) and detectable BX concentrations can persist after ensiling (Gross et al. 2023). Therefore, future studies should determine whether variation in BX concentrations affects microbial community structure, fibre and starch fermentation, volatile fatty acid profiles, nitrogen metabolism or host‐related physiological responses in ruminants, particularly under higher insect pressure or in maize genotypes with contrasting BX profiles.

## 4 Conclusions

In conclusion, early and mid‐early forage maize genotypes reached comparable physiological maturity at harvest, resulting in similar nutrient composition, forage quality and BX concentrations. The BX metabolites were associated with insect pressure; however, damage level was low and BX concentrations were not negatively related to forage nutritive value. These findings indicate that plant defence capacity and high forage quality can co‐occur under practical growing conditions. Future studies should investigate the functional role of BX under higher insect pressure and test, whether variation in BX concentrations is relevant for ruminal fermentation or host‐related physiology.

## Ethics Statement

The present study was performed without the experimental use of animals or animal‐derived products.

## Data Availability

The data that support the findings of this study are available from the corresponding author upon reasonable request.
